# Thymus and activation-regulated chemokine (CCL17) as a clinical biomarker in atopic dermatitis: significance and limitations in the new treatment era

**DOI:** 10.3389/falgy.2024.1473902

**Published:** 2025-01-23

**Authors:** Yoko Kataoka

**Affiliations:** Department of Dermatology, Osaka Habikino Medical Center, Habikino, Osaka, Japan

**Keywords:** atopic dermatitis, monitoring biomarker, thymus and activation-regulated chemokine, biologics, JAK inhibitors, topical corticosteroid

## Abstract

Thymus and activation-regulated chemokine (TARC; CCL17) is a T-helper-2 chemokine that reflects atopic dermatitis (AD) disease activity. Since 2008, serum TARC levels have been commercially measured in Japan, and clinical experience has shown the usefulness of TARC. The fallacy that eczema is always visible often hinders successful treatment, when there is subclinical inflammation which is inferable from the TARC level. AD treatment has entered a new era with higher therapeutic efficacy. TARC has a different meaning than it did previously, and its significance and limitations are discussed. First, a more appropriate topical therapy monitoring TARC would be useful in selecting truly necessitated patients for expensive new therapies. Dupilumab quickly lowers serum TARC before clinical improvement, and its normalization is not a criterion for dose reduction. However, in some severe cases, TARC may help determine whether to continue treatment. During treatment with JAK inhibitors, serum TARC levels are often elevated and may be abnormally high, leading to the exacerbation of dermatitis. Prurigo nodularis is divided into two types associated with elevated and normal TARC levels, which may aid in the selection of therapeutic agents. In this new era, TARC remains a useful biomarker for more accurate drug selection and the determination of therapeutic efficacy; Currently, in clinical trials of AD, all outcome measurements depend on the clinical score; however the use of a biomarker, such as TARC, as a secondary outcome measure will clarify the characteristics of each drug and the pathophysiological conditions for which it is expected to be effective.

## 1 Introduction

Atopic dermatitis (AD), a chronic pruritic dermatosis, confers a significant disease burden. In the past decade, increased availability of many molecular-targeted therapies, with remarkably improved therapeutic outcomes. In this era of novel, expensive therapies, precision medicine requires biomarkers for classifying and identifying patients for whom specific therapies are suitable (1, 2). For monitoring disease activity, the thymus and activation-regulated chemokine (TARC; also known as CCL17) is the most supported biomarker (3), because of its T helper 2 cell-mediated chemotactic activity (4). Type 2 inflammatory cells play a crucial role in AD pathogenesis (5). Although serum TARC levels are closely related to AD clinical disease activity (6, 7) their usefulness is not widely recognized because of the need for laboratory-based measurement. In Japan, serum TARC measurements have been commercially available since 2008, which, we realized, improves AD treatment outcomes (8). In AD treatment, the recent introduction of many biological agents and JAK inhibitors (JAKIs) has induced changes in TARC levels that differ from those during conventional topical therapy and may warrant an understanding of the different underlying mechanisms. Based on the 15-year clinical experience with TARC monitoring in Japan, we discuss the significance of TARC as a monitoring marker, before and after the introduction of molecular-targeted therapies. In this article serum TARC levels were measured by a chemiluminescent enzyme immunoassay using the HISCL® system (Sysmex, Hyogo, Japan) and a TARC Assay Kit (Shionogi, Osaka, Japan) whose detection scope is 10–30,000 pg/mL.

## 2 Case description which implies key learning points from TARC monitoring during conventional treatment of AD

A 10-year-old girl, originally from a European country, presented to our hospital with generalized dermatitis that had persisted since early infancy. For marked, refractory lesions, she was prescribed whole-body application of emollient, twice a day, and a 5-day course of very-high-potency topical corticosteroids (TCS) and showed transient improvement. However, within a few days of switching to only emollient or moderate TCS, AD flared up, with nocturnal pruritus that disturbed sleep. On her first visit to our department, she presented with generalized erythema, excoriations, and a serum TARC level of 2,175 pg/mL, for which she was prescribed whole-body (excluding the face) high-potency TCS application for 1 month that resulted in symptom disappearance and normalization of the serum TARC to 262 pg/mL. The patient was instructed on TCS weaning off before leaving for her country. Upon her return to Japan 4 months later, she had no skin lesions on semi-weekly proactive TCS treatment, with the TARC level maintained at 250 pg/mL. At the 1-year follow-up, all skin lesions, except for a small lesion, had cleared up with weekly TCS application, with normal TARC level (379 pg/mL). The patient was extremely grateful that her skin had remained normal for a year, without any problems in her daily life, and was hopeful about her future.

**Figure 1:**
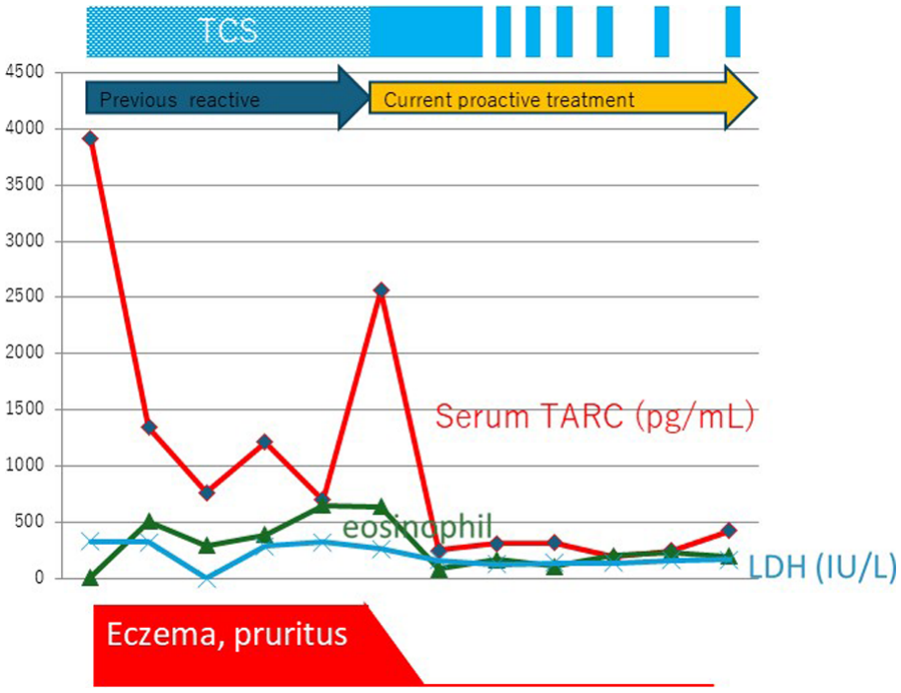
The truth of waning and waxing atopic dermatitis. A representative case of adult atopic dermatitis after successful proactive topical corticosteroid treatment. Changes in the serum thymus and activation-regulated chemokine (TARC) levels indicate that an appropriate method of application is important for achieving successful outcomes. Eo, eosinophils; IgE, immunoglobulin E; LDH, lactate dehydrogenase; TCS, topical corticosteroid.

Despite using similar drugs, differences in treatment methods can frequently alter the outcomes drastically, even among Japanese residents. Figure 1 shows the changes in the biomarkers in a representative case. The treatment methods differ depending on whether the TCS dosage was guided by TARC monitoring.

### 2.1 Truth of waning and waxing (flare-ups) AD

Both physicians and patients believe that AD is a chronic disease with recurrent exacerbations; however, in many cases, these exacerbations are iatrogenic and caused by high or low drug doses.

### 2.2 Accurate proactive treatment for better outcome

A meta-analysis demonstrated the robust superiority of proactive treatment (9), although the preliminary findings are too nascent for clinical implementation, given the diverse disease severities and dermatitis disease activities among AD patients. Nonetheless, a precise treatment plan based on these two factors will improve outcomes. Abnormally high TARC levels indicate accelerated type 2 inflammation wherein early intensive therapy for rapid mitigation of inflammation predicts a better prognosis with careful drug/dose reduction to prevent flare-ups. Objective improvement and Patient Reported outcome (PRO) are insufficient to determine whether the current treatment adequately controls inflammation. The sustained normalization of TARC levels indicates good long-term control (10) and validates AD treatment. However, a weak or insufficient TCS regimen that does not reduce TARC levels (11), and short-term treatment will cause repeated flare-ups. Residual inflammation, even if occult, will inevitably flare-up after treatment discontinuation. Long-term control can be achieved by controlling residual subclinical inflammation (12).

### 2.3 Re-recognition of the importance of topical therapy

With accurate proactive treatment and TARC-monitoring-based tight control, good control of type 2 inflammation is achievable with topical therapy alone in a significant proportion of patients, with long-term remission maintained by administering minimal, safe doses of topical drugs. However, in cases with TARC levels refractory to initial intensive TCS or an increase in TARC levels with post-stabilization TCS reduction, local therapy alone is potentially ineffective, and systemic therapy should be considered.

### 2.4 Goal of AD treatment and patient adherence

Unlike in other countries, in Japan, the AD guidelines clearly state the treatment goal: “The goal of treatment is to reach and maintain a state in which symptoms are absent or mild with minimum drug. Even when this goal is not attained, the objective is to maintain a clinical state with mild symptoms and without rapid exacerbations that affect daily activities” (13). These guidelines were developed to mitigate the confusion regarding AD treatment in Japan in the 1990s (14) that significantly reduced the quality of life of many patients. Then, TCS was considered merely a symptomatic treatment, with a lack of clear physician-led guidance on the dosage or cessation of topical application, which was left to the patient's discretion and, consequently, many patients experienced repeated flare-ups despite TCS usage. Misleading media reports about adverse effects rapidly increased the number of patients with steroid avoidance-induced severe dermatitis and led to significant reduction in the quality of life. This social problem was mediated not only by patients’ anxiety about side effects, but also a discordance between patients and physicians regarding treatment goals, as patients did not want reactive management of recurrent flares with TCS but aimed to achieve long-term control. However, at that time, many dermatologists did not believe that this goal was achievable and considered flare management as the primary objective of pharmacotherapy. In Japan, since 2008, TARC monitoring aided precise disease control, and the number of steroid-phobic patients has decreased. For the successful treatment of chronic disease, both the physician's treatment strategy and the patient's adherence are essential, and TARC monitoring provides a numerical treatment goal to aid both (7).

## 3 Significance of serum TARC monitoring with newer AD treatments

### 3.1 Biologics

**Figure 2:**
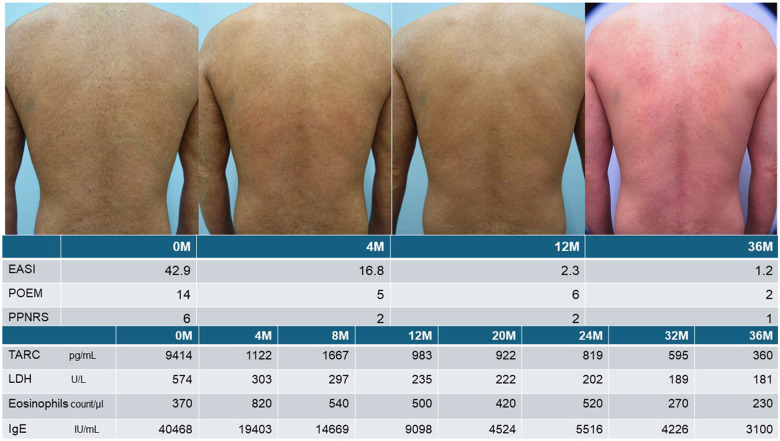
Usefulness of TARC monitoring during dupilumab treatment in patients with severe AD. Dupilumab treatment was initiated for a middle-aged patient with severe refractory AD and, because dupilumab strongly suppresses type 2 inflammation, serum TARC levels decreased rapidly and significantly within 4 months but remained abnormal. TARC levels increased, at the 8th month since dupilumab initiation, after cessation of cyclosporine treatment following improvements in subjective and objective symptoms, which suggests that cyclosporine levels should be tapered after confirming the effect of dupilumab in patients with severe disease who were previously treated with cyclosporine. At the 12th month, the subjective and objective symptoms had improved, but the TARC level was abnormal. Despite dupilumab's ability to suppress type 2 inflammation, presumably, the inflammation persisted as indicated by the higher-than-normal TARC levels. After confirming TARC normalization at the 32nd month, the dosing interval was extended to 3 weeks, and the skin recovered to an almost normal state without relapse, despite the longer inter-dose interval.

In patients who are appropriately treated with conventional therapies, the serum TARC level normalizes, and the treatment goal can be achieved by maintaining normal TARC levels when tapering pharmacotherapy. Newer molecular-targeted drugs alter serum TARC levels differently: dupilumab (15), tralokinumab (16), and lebrikizumab (17) decrease serum TARC, and dupilumab has potent TARC-normalizing capability whereby TARC levels normalize within a few months of initiation. During early treatment, despite normal TARC levels, many patients may have decreased but persistent subjective and objective clinical symptoms. Therefore, TARC normalization precedes clinical improvement, and does not necessarily imply the disappearance of subclinical inflammation. Alternatively, in some refractory cases, TARC levels decline after starting dupilumab although TARC normalization may require continued treatment for a few years. In real-world settings, although good control is often achieved with extended-dosing intervals of dupilumab (18), early dosing-interval extension in severe cases confers a risk of relapse; in such cases, TARC monitoring may aid decision-making (Figure 2). Although abnormally high TARC levels despite prolonged dupilumab treatment may indicate dupilumab resistance, TARC is not a predictor of the response to dupilumab (19).

Nemolizumab, an IL31RA antibody, has proven efficacy in AD-induced pruritus, but may increase serum TARC levels. In clinical trials, TARC elevation was unassociated with changes in the EASI scores (20). However, in some patients, nemolizumab is associated with severe new-onset or worsening dermatitis, with abnormally high TARC levels, that warrants TCS intensification or nemolizumab discontinuation.

### 3.2 JAKIs

Three JAKIs—baricitinib, upadacitinib, and abrocitinib—are currently used for systemic treatment of AD and, despite a good clinical response, increase TARC levels during prolonged treatment (>6 months) (21), which detracts from TARC's value as a clinical biomarker (22). Nevertheless, this phenomenon is noteworthy because of the parallel increases in serum immunoglobulin E and TARC levels that suggest enhanced type 2 inflammation.

## 4 Discussion

Approximately 55% of adults with moderate-to-severe AD have inadequate disease control (23). TARC aids efficacy maximization and identification of the limitations of conventional therapies; however, newer therapies may be unsuitable for some patients with moderate-to-severe disease, and conventional treatments should be optimized before initiating expensive newer therapies (24) that some patients would not prefer. In some countries, national financial allocations preclude the provision of expensive drugs. It is imperative to prevent the denial of appropriate medical care to poor patients. The high cost of AD treatment is an important social cost, and a clinical study to identify patients who would benefit from cyclosporine has been planned (25). Serum TARC monitoring facilitates optimization of inexpensive conventional therapies and patient selection for new therapies.

Although a systematic review confirmed the importance of remission induction and maintenance (11), the extent of posttreatment remission is unclear. The suppression of inflammation induces not only symptomatic improvement but also TARC reduction and decreased type 2 inflammation, which could theoretically indicate normalization. Although yet unsupported by clinical evidence, based on numerous clinical experiences, it is recommended that the serum TARC levels should be maintained below 600 pg/mL in patients who are older than 6 years. Recently, steroid withdrawal has become a problem in Europe and the U.S. (26, 27). By rejecting the dogma that AD is incurable, physicians and patients can establish treatment goals based on objective targets defined by numerical data.

The dissociation of TARC changes from skin manifestations during biologic or JAKI treatment is a remarkable phenomenon. Recently, in Japan, there have been several cases, albeit unpublished, of clinical secondary ineffectiveness indicated by preceding elevated TARC levels during continuous JAKI administration. Proteomics of lesional skin revealed decreased levels of various chemokines, including TARC, soon after administration (28). It is unclear whether local and systemic responses differ or long-term administration induces different changes; however, elevated serum TARC levels are potentially associated with subsequent worsening of AD. Studies examining real-world biomarkers over time have significant implications, and follow-up rates are 70% for dupilumab; 21% for tralokinumab; and 19%, 35%, and 19% for baricitinib, upadacitinib, and abrocitinib (21), respectively. A detailed comparison of the evolution of TARC and clinical scores, including dropout cases, may help stratify real-world responders.

Prurigo nodularis (PN) is a refractory chronic skin disease, with a high disease burden on conventional treatment. New therapies have been developed based on PN pathophysiology, which mainly involves type 2 inflammation, pruritic neurotransmission, epidermal thickening, and dermal fibrosis (29). The efficacy of dupilumab (30) against type 2 inflammation and nemolizumab (31) against IL31, which mediates itch transmission, has been confirmed in clinical trials. The association of PN with atopy in a subset of patients fosters an assumption that PN is an AD subtype, whereas PN can occur without atopy. Important disease-specific differences between PN and AD have recently been reported (32, 33). Nemolizumab demonstrated clear efficacy in PN and rapidly improved pruritus, although one of its most common adverse events was AD (28). IL31 may have unique effects on the immune and nervous systems (34), including negative regulation of TH2 cells or CGRP-mediated activation of immunoregulatory pathways in sensory neurons. Distinguishing between classical PN without AD which is super-responder to nemolizumab and PN + AD could facilitate the selection of an effective treatment without worsening AD. Serum TARC levels are nearly normal in classical PN without dermatitis, whereas it is frequently elevated in AD-associated PN. Thus, TARC may constitute a pretreatment stratification marker, such as for dupilumab in patients with PN and high serum TARC, because of potentially enhanced type 2 inflammation, and selection of nemolizumab in patients with PN and normal serum TARC.

To evaluate the efficacy and stability of novel therapeutics over time based on biological mechanisms, TARC and other biomarkers are good tools. Currently for clinical trials, Harmonizing Outcome Measures for Eczema (HOME) recommends four core outcome measures: EASI for clinical signs, POEM and PPNRS as subjective symptoms, DLQI for quality of life, and ADCT or RECAP (35) for long-term control. However, the current practice of using expensive drugs without predicting their efficacy must be changed, possibly by disclosing biomarker trends as secondary outcomes in clinical trials, with stratified biomarker analyses, wherein serum TARC is a leading candidate for monitoring type 2 inflammation.

Drug breakthroughs have ushered in a paradigm shift in the therapeutic goals for several chronic inflammatory diseases, led by rheumatoid arthritis (RA). Highly effective biologics have redefined the goals and strategies of RA therapy whereby patient outcomes have improved dramatically. Furthermore, in RA, not only novel drug development but also treatment strategy reevaluation has improved outcomes. A review of Treat to Target (T2T) studies for RA revealed the superiority of a tight control strategy over a specific drug to control RA. A T2T approach targeting remission or low disease activity is achievable in early RA with less expensive, rather than newer, drugs (36). Adjunctive serum TARC monitoring, with clinical findings, enables precision medicine for tight control. However, in many cases, iatrogenic symptom relapse secondary to dosage reduction based on inaccurate judgments may occur because biomarkers are not monitored. Thus, even with the same drug, there is a possibility that treatment efficacy can be improved by revising treatment strategies.

Recently, remission has been also included as a treatment goal for asthma, which reflects a paradigm shift from short-term symptom control to long-term symptom prevention. An international expert panel presented a framework for complete remission in asthma patients, on and off treatment. Complete remission is defined as clinical remission plus objective resolution of asthma-related inflammation, including biomarker monitoring (37). For AD, a T2T-based conceptual approach was recently proposed (38–40), and recommended that patient satisfaction, with minimal impact on the quality of life and clear/almost-clear skin with no or minimal itching, should be the ultimate treatment goal in AD. Unfortunately, clinical remission, complete remission, and strategies to achieve these outcomes have not been described. Serum TARC is a potential candidate marker for objectively ascertaining the resolution of AD-related inflammation during AD remission. Currently, the target achievement for T2T in AD is limited to clinical scores and PROs. The addition of biomarkers will brighten the goal landscape.

## 5 Limitations

TARC elevations occur in various autoimmune (41), skin diseases (8, 42) and Hodgkin's lymphoma (43). Thus, TARC is not a diagnostic marker for AD alone. Some patients with persistent lichenified lesions have relatively low serum TARC levels. TARC levels were generally higher in younger children (13). The recommendation for targeting TARC levels is empirical and not based on randomized controlled trial evidence.

## 6 Conclusion

In the new era of AD treatment, clinical and complete remission should be defined, and a consensus on the goals of AD treatment and strategies to achieve them should be established. To evaluate the effectiveness of a drug, objective and precise measurements, including biomarkers, should be considered, besides conventional evaluations. When selecting an expensive new treatment in clinical practice, monitoring biomarkers, such as TARC, optimizes conventional treatment and patient selection and facilitates the evaluation of therapeutic efficacy in the context of the patient's pathology.

## Data Availability

The original contributions presented in the study are included in the article/Supplementary Material, further inquiries can be directed to the corresponding author.
